# How HTLV-1 may subvert miRNAs for persistence and transformation

**DOI:** 10.1186/1742-4690-5-101

**Published:** 2008-11-12

**Authors:** Amel B Bouzar, Luc Willems

**Affiliations:** 1 Molecular and Cellular Biology lab of the Gembloux Agricultural University (FUSAG)n°13, avenue Maréchal Juin, 5030 Gembloux, Belgium; 2 Molecular and Cellular Epigenetics, Interdisciplinary Cluster for Applied Genoproteomics (GIGA) of University of Liège (ULg) avenue de l'Hôpital n° B34, Sart-Tilman, 4000 Liège, Belgium

## Abstract

Distinct mechanisms are used by viruses to interact with cellular miRNAs. The role of microRNAs in viral replication and persistence ranges from viral-encoded microRNAs to suppressors of RNA interference. Viruses can also exploit cellular miRNAs for influencing cellular metabolism to ensure efficient replication or latency. In particular, two recent studies provide examples of how HTLV-1 may co-opt or subvert cellular miRNAs for persistent replication and oncogenic purposes. The pathways modulated by these described miRNAs are critically involved in apoptosis, proliferation and innate immune response.

## Biogenesis of miRNAs

MicroRNAs are initially transcribed by RNA polymerase II as a primary miRNA (pri-miRNA) transcript and processed in the nucleus by RNase III enzyme Drosha and its cofactor DGCR8 [1-4]. Cleavage of the pri-miRNA by the Drosha-DGCR8 heterodimer generates a 60–70 nucleotide precursor miRNA (pre-miRNA), which is then transported to the cytoplasm by the nuclear export factor exportin 5 and GTP-bound Ran. Cytoplasmic pre-miRNA is further recognized by RNase III enzyme Dicer bound to its cofactor TRBP, and the pre-miRNA is cleaved into a mature miRNA. Finally, loading of the miRNA into the RNA-induced silencing complex (RISC) allows the miR-RISC to target cognate mRNA via imperfect base complementarity. miR-RISC can induce the cleavage of or may inhibit the translation of targeted mRNAs.

There is some promiscuity amongst the several hundreds of cellular miRNAs, each miRNA can potentially target many discrete mRNAs, thereby modulating a broad spectrum of biological functions. Indeed, RNA interference is a major mechanism used to control viral infections in plants and invertebrates [4]. As a counter play to the cell's RNAi, viruses encode suppressors of RNA silencing, which target several key steps in the RNAi process [5,6]. Compelling evidence that this process also applies to mammalian cells and viruses is presently accumulating in the literature [7].

## Biological roles of virus-encoded non-coding RNAs

The first evidence of virus-encoded miRNAs emerged from studies of Herpesviruses (e.g. Epstein-Barr virus encoded microRNAs [8,9]). These viral miRNAs exert a wide variety of functions ranging from stimulation of proliferation, inhibition of apoptosis, maintenance of latency, regulation of immune response and cellular metabolism. For example, miR-I encoded by the herpes simplex virus 2 (HSV 2) has a critical role in neurovirulence through modulation of ICP34.5 expression [10]. On the other hand, miR-K12-10 encoded by Kaposi sarcoma-associated herpesvirus (KSHV) plays a key role in cellular transformation through downregulation of Kaposin mRNA translation [9,11]. Non-herpesviruses such as Simian Virus 40 (SV40) can also encode a miRNA which reduces the susceptibility of infected cells to lysis by cytotoxic T cells, allowing the virus to evade the host immune response [12]. Additionally, adenovirus VA1 noncoding RNA is not a virus-encoded miRNA, but is able to subvert the cell's RNAi pathway by competing with cellular pre-miRNAs for exportin-5 in the nucleus as well as by binding Dicer in the cytoplasm [13]. VA1 RNA also interacts with PKR (interferon-inducible double-stranded RNA-dependent protein kinase), thereby blocking its activation and the subsequent phosphorylation of eukaryotic translation-initiation factor 2α (eIF2α).

## Viruses can be targeted by cellular miRNAs

Given the prevalence of miRNA genes and the short six-nucleotide seed pairing that is needed to establish miRNA-mRNA interactions, viral genomes are also likely targets of human cellular miRNAs [14]. Initial evidence supporting this idea was illustrated by the binding of the host-cell's miRNA miR-32 to a site in primate foamy virus type 1 (PFV-1) RNA, which restricted viral RNA accumulation [15]. The PFV-1 Tas protein counteracted this mechanism and functioned as a silencing suppressor to relieve this repression and allowed PFV-1 replication.

Surprisingly, the interaction of viral genomes with cellular miRNAs may also promote rather than inhibit viral replication. Thus, the binding of liver-specific miR-122 to the 5' end of hepatitis C virus (HCV) RNA increased viral RNA levels, probably owing to a stimulation of viral replication or to the re-localization of viral RNA [16,17]. Interferon-beta (IFNβ) treatment reduced miR-122 but increased the expression of miR-1/miR-30/miR-128/miR-196/miR-296/miR-351/miR-431/miR-448 miRNAs [18]. Similarly, the introduction of synthetic miRNA mimics corresponding to these 8 miRNAs into cells reproduced the antiviral effects of IFNβ on HCV replication. Conversely, neutralization of these miRNAs reduced the antiviral effects of IFNβ against HCV. Moreover, inoculation of miR-122 antisense oligonucleotides into mice resulted in the inhibition of cholesterol biosynthesis and HCV replication [19,20].

The ability of mammals to regulate retroviruses like HIV-1 has been intensely debated [3,21]. Recent evidence that HIV-1 replication can be promoted by lowered expression of Dicer and Drosha supports a role of the miRNA silencing machinery in controlling viral infection [22]. In fact, the 3' ends of HIV-1 mRNAs are targeted by a cluster of cellular miRNAs (miR-28, miR-125b, miR-150, miR-223 and miR-382), which inhibit HIV-1 protein translation and viral production [23]. Since these miRNAs are upregulated in resting CD4+ T cells, the RNAi machinery may also contribute to viral latency. Given that specific inhibitors of these miRNAs substantially counteract their effects on the target mRNAs, RNA interference may potentially be useful for purging the HIV-1 reservoir of latent virus [24].

## Targeting of cellular miRNAs by HTLV-1

Viruses can also exploit cellular miRNAs for influencing cellular metabolism, proliferation, apoptosis and, ultimately, transformation [2]. For example, the Epstein Barr virus (EBV) infection of human B lymphocytes increases miR-155 [25] and miR-146a [26] expression through a mechanism that, at least in part, involves latency membrane protein 1 (LMP1). Similarly, increased expression of the BIC/miR155 and other oncogenic miRNA transcripts in animal lymphomas due to retroviral integrations have also been documented [27-29]. However, despite the few examples of subversion of cellular miRNA by viruses, the field of viral oncogenesis mediated through miRNA-expression remains insufficiently explored. The papers by Pichler et al. [30] and by Yeung et al. [31] represent the first reports linking Human T-lymphotropic virus type 1 (HTLV-1) infection with the modulation of human miRNA expression.

HTLV-1 infects about 10–20 million people worldwide and, in a significant proportion of them (~2–4%), causes either adult T cell leukemia (ATL) or HAM/TSP (HTLV-associated myelopathy/Tropical Spastic Paraparesis). HTLV-1 infects and replicates in CD4+ and CD8+ T lymphocytes [32] as well as in dendritic cells [33]. HTLV-1 persistence and replication critically involves the virus-encoded Tax protein [34,35]. Amongst a broad variety of functions, Tax activates transcription of viral and cellular genes (e.g. TNF-α), accelerates cell cycle progression, interferes with apoptosis, inhibits checkpoints and induces DNA damage. To carry out these functions, Tax interacts with and modulates the activity of more than 100 cellular proteins (recently reviewed by Boxus et al [36]). The new findings from Pichler et al. and Yeung et al. add another level of complexity by identifying miRNAs as being either directly activated by Tax or associated with HTLV-1 induced cell transformation.

The approaches used to narrow down the spectrum of candidate miRNAs in the two studies were different. Pichler et al. selected a limited number of miRNAs having links with cancer and being overexpressed in regulatory T lymphocytes. Their rationale for miRNA selection was that the phenotype of Tregs resembles that of ATL cells. RT-PCR quantification identified upregulated (miR-21, miR-24, miR-146a, miR-155) and repressed (miR-223) miRNAs in a series of cell lines derived from ATL patients, HAM/TSP patients and HTLV-1 or Tax transformed cells. Expression of one of these, miR-146a, was directly activated by Tax through the proximal NF-κB site of the MIRN146A gene promoter.

Yeung et al. profiled 327 human miRNAs in 7 HTLV-1 transformed cell lines and 4 PBMC samples from acute ATL patients. Among 15 miRNAs whose expression was consistently modified compared to paired controls, only 3 (miR-93, miR-130b and miR-18a) were also induced upon the activation of normal PBMCs with phorbol myristate acetate. The authors then confirmed the differential expression of miR-93 and miR-130b using qRT-PCR. Computational analysis and luciferase reporter assays demonstrated that the p53-inducible tumor suppressor protein (TP53INP1) was a target shared by both miR-93 and miR-130b. Consistently, antagomirs for miR-93 and miR-130b (used to knock down the level of miR-93 and miR-130b in cells) restored TP53INP1 expression and increased the apoptosis of HTLV-1 transformed MT4 cells. Conversely, siRNA knock-down of TP53INP1 rescued MT4 from cell death induced by the miR-93 and miR-130b antagomirs. Increased expression of miR-130b occurs at least partly through transcriptional activation by Tax. Finally, Yeung et al. also reported a series of miRNAs that are repressed in ATL cells.

## HTLV-1 upregulates miRNAs involved in proliferation, apoptosis and immune response

**Figure 1:**
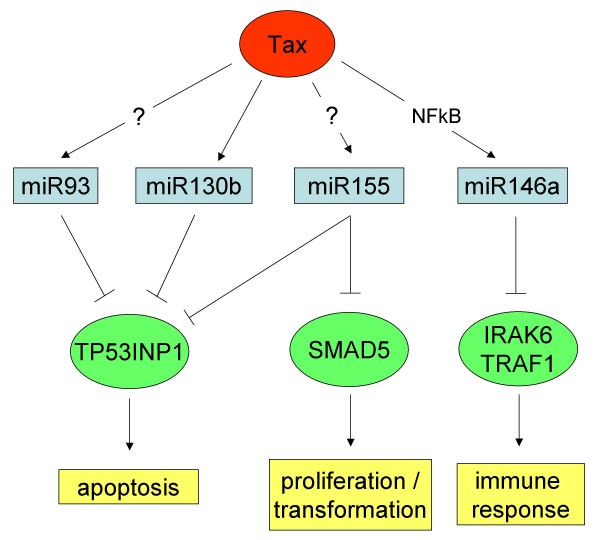
**Overview of 4 miRNAs overexpressed in ATL cells and their targeted mRNAs: TP53INP1, Smad5 and IRAK6/TRAF1.** These genes are critically involved in pathways important for viral persistence and oncogenicity. Tax directly transactivates the miR-146a (through a NF-κB site) and miR-130b promoters. Only one of these pathways Tax→miR130b→TP53INP1→apoptosis has presently been demonstrated functionally.

Although technical procedures and methodological approaches were different, several identical miRNA changes were identified in both studies. Given the complexity of the biological processes and the reported datasets, we focus in figure 1 on a selected number of relevant miRNAs reported in the two studies that are overexpressed in ATL (miR-93, miR-130b, miR-155 and miR-146a). These miRNAs target genes (TP53INP1, SMAD5, IRAK6/TRAF1) are involved in apoptosis, cell proliferation or transformation, and the regulation of immune response.

A first salient outcome from the Pichler and Yeung reports is that HTLV-1 directly modulates the expression of at least two miRNAs by transactivating the miR-130b and miR-146a promoters. These data show examples of how viruses may directly modulate the transcription of cellular miRNAs, possibly to favor replication and/or oncogenicity. Considering that the HTLV-1 Tax protein is known to activate a very long list of genes through NFκB/CREB/SRF [35], it is expected that additional miRNAs could be directly modulated by Tax, through the transactivation of their cognate promoters. We can also speculate whether other steps in the biogenesis of miRNA may be under the control of HTLV-1 encoded proteins, such as Rex for nuclear export or NC for encapsidation. Further investigations are definitely required to test these predictions.

A second striking observation is that a single transcript (i.e. TP53INP1) is targeted by 3 different miRNAs (miR93, miR130b and miR155) that are overexpressed in ATL cells. TP53INP1, whose transcription is activated by p53, induces cell cycle arrest in G1 and enhances p53-mediated apoptosis [37]. The role of TP53INP1 in oncogenesis has been reported in a series of models. In pancreatic cancer, TP53INP1 is repressed by miR-155, and its restoration inhibits tumor development [1]. Growth of colorectal tumors is exacerbated in TP53INP1-deficient mice [38]. TP53INP1's expression is reduced during the development of breast cancer [39], gastric cancer [40], pancreatic cancer [1] and melanoma [41], but curiously increased in thyroid cancer [42]. The convergence of multiple miRNAs onto a single target reinforces the idea that TP53INP1 could also be a relevant factor for HTLV-1 leukemogenesis.

Besides its effects on TP53INP1, miR155 also targets genes critically involved in cellular proliferation and transformation. In fact, higher expression of miR-155 has been exemplified in several types of hematopoietic malignancies including B-cell and Hodgkin's lymphoma [4,43,44]. Overexpression of miR-155 causes myeloproliferative disorders or B cell lymphoma in mouse models. Mice lacking miR-155 exhibit defective humoral responses after immunization, consistent with a specialized function for miR-155 during infection [45,46]. In T lymphocytes, miR-155 regulates T cell lineage fate by promoting T helper type 1 (Th1) versus T helper type 2 (Th2) differentiation. BIC/miR-155 is overexpressed in Treg cells, consecutive to Foxp3-induced promoter activation. Consistent with its association with an activated T cell phenotype, miR-155 is absent from the HIV-1 latent reservoir [23]. miR-155 is also involved in lymphocyte activation and the latency of Epstein-Barr virus (EBV)-infected cells [47]. miR-155 expression in EBV-infected cells is dependent on NF-κB signaling and requires a conserved AP-1 element in the miR-155 promoter. Using luciferase reporter systems, Smad5 was confirmed as a gene under the control of miR-155 [47]. Smad5 is one of the 5 receptor-regulated Smads which orchestrate transforming growth factor β (TGFβ) signaling. TGFβ induces miR-155 expression and promoter activity through Smad4 [48]. Conversely, the knockdown of miR-155 suppresses TGFβ-induced cell migration and invasion [49]. The involvement of miR-155 in ATL further complicates the interplay between HTLV-1 and the TGFβ pathway [50-52] but underscores its critical role in transformation.

Another key player overexpressed in HTLV-1-infected ATL cells is miR-146a. This miRNA was first identified as an immune system regulator induced by lipopolysaccharide (LPS) and proinflammatory cytokines (such as interleukin 1 and tumor necrosis factor) [53,54]. miR-146 is among the most highly expressed miRNAs in regulatory T cells. Compared to Th2 and naïve T cells, miR-146 levels are higher in Th1 cells. Amongst the growing list of confirmed target genes of mir-146, IRAK1 and TRAF6 adaptor molecules are essential for Toll-like receptor and interleukin 1 receptor signaling [55,56]. Reduction of IRAK1 and TRAF6 expression might therefore be involved in the regulation of the innate immune response.

## Conclusion

**Figure 2:**
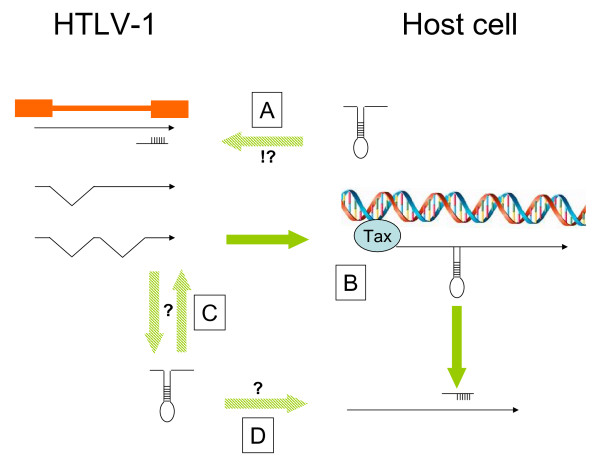
**Confirmed and hypothetical (?) interplays between HTLV-1 and virus or cell-derived small non-coding RNAs. **A. Given the imperfect base-pairing requirements, the multiplicity of cellular miRNAs and the length of the proviral genome, it seems likely (!?) that HTLV-1 is targeted by cellular miRNAs. B. HTLV-1 Tax directly transactivates cellular miRNA genes. Expression of a small non-coding viral RNA by HTLV-1 could affect viral (C) or cellular (D) gene expression through hypothetical (?) mechanisms of RNA interference.

HTLV-1 has not yet been shown to encode a viral miRNA, although potential candidate genes such as Hbz [57,58] have not been sufficiently evaluated. In this context, it is intriguing that the Hbz RNA, but not its encoded proteins, promotes T cell proliferation [59]. Could this activity be through an RNA-mediated mechanism similar to RNA interference? The papers by Pichler et al [30] and Yeung et al. [31] demonstrate how HTLV-1 modulates a series of miRNAs, although only a few of them are commented on here. What has not been mentioned here but should be stressed is that a number of miRNAs are repressed in ATL cells such as let-7 (a tumor repressor of Ras and Myc transformation) or miR34b (inducing cell cycle arrest), further amplifying the complexity of HTLV-1's interaction with miRNAs in the transformation pathways. In another perspective, it could also be considered that HTLV-1 persists in a limited population of cells characterized by a given miRNA profile. This view of opportunistic viral persistence has recently been illustrated for how HIV-1 latently infects resting CD4 T-cells [23]. Further investigations of confirmed and speculative interplays between HTLV-1 and small non-coding RNAs (figure 2) are merited.

## Competing interests

The authors declare that they have no competing interests.

## Authors' contributions

AB and LW collected data from the literature and wrote the paper. All authors read and approved the final manuscript.
